# Selective particle attention: Rapidly and flexibly selecting features for deep reinforcement learning

**DOI:** 10.1016/j.neunet.2022.03.015

**Published:** 2022-06

**Authors:** Sam Blakeman, Denis Mareschal

**Affiliations:** a Sony AI, Wiesenstrasse 5, 8952, Schlieren, Switzerland; b Centre for Brain and Cognitive Development, Department of Psychological Sciences, Birkbeck, University of London, Malet Street, WC1E 7HX, United Kingdom

**Keywords:** Selective attention, Visual features, Reinforcement learning, Particle filter, Neural networks

## Abstract

Deep Reinforcement Learning (RL) is often criticised for being data inefficient and inflexible to changes in task structure. Part of the reason for these issues is that Deep RL typically learns end-to-end using backpropagation, which results in task-specific representations. One approach for circumventing these problems is to apply Deep RL to existing representations that have been learned in a more task-agnostic fashion. However, this only partially solves the problem as the Deep RL algorithm learns a function of all pre-existing representations and is therefore still susceptible to data inefficiency and a lack of flexibility. Biological agents appear to solve this problem by forming internal representations over many tasks and only selecting a subset of these features for decision-making based on the task at hand; a process commonly referred to as selective attention. We take inspiration from selective attention in biological agents and propose a novel algorithm called Selective Particle Attention (SPA), which selects subsets of existing representations for Deep RL. Crucially, these subsets are not learned through backpropagation, which is slow and prone to overfitting, but instead via a particle filter that rapidly and flexibly identifies key subsets of features using only reward feedback. We evaluate SPA on two tasks that involve raw pixel input and dynamic changes to the task structure, and show that it greatly increases the efficiency and flexibility of downstream Deep RL algorithms.

## 1 Introduction

Deep Reinforcement Learning (RL) has emerged as a core framework for training agents to solve complex decision-making tasks, such as playing video games from raw pixel values (Mnih et al., 2016, Mnih et al., 2015). Despite these successes, Deep RL is often criticised for being data inefficient and inflexible to changes in task (Lake, Ullman, Tenenbaum, & Gershman, 2017). This is largely due to the fact that representations are most commonly learned in an end-to-end manner via backpropagation. The learned function therefore depends on all the input values and the intermediate representations are highly tuned to a specific task, which reduces both efficiency and flexibility. One way to overcome these issues is to apply Deep RL to representations that have been learned from the raw input in a more task-agnostic manner, such as via unsupervised learning (Bengio, 2012) or additional training objectives (Jaderberg et al., 2016). This helps to improve efficiency because the dimensionality of the pre-learned representations is often lower than the original input. Flexibility is also improved because the pre-learned representations are not tuned to a specific task and can therefore support a range of different tasks. These improvements are limited however, because all pre-learned features are provided as input to the Deep RL algorithm. The scale of the dimensionality reduction is restricted by the number of pre-learned representations and changes to the task will require backpropagating errors across all of the pre-learned features, thereby over-writing any previously learned function.

We propose that these limitations of using pre-learned representations can be overcome by applying a selective attention mechanism that is inspired by biological agents. Selective attention in biological agents acts as a gating mechanism, whereby the firing of neurons that encode an attended feature increase, while the firing rates of neurons encoding a non-attended feature decrease (Saenz et al., 2002, Treue and Trujillo, 1999). This gating mechanism helps to improve the efficiency and flexibility of the learning algorithm in several ways. Firstly, it serves to further reduce the dimensionality of the learning problem (Jones and Canas, 2010, Niv et al., 2015, Wilson and Niv, 2012), which increases the efficiency of learning and reduces sensitivity to changes in the environment. Secondly, if the task or goal changes then a new subset of features can be chosen quickly and flexibly without over-writing the newly learned function thereby alleviating catastrophic forgetting and interference. Finally, selective attention can allow for learning over different time-scales. For example, the underlying representations can be learned slowly (Schyns, Goldstone, & Thibaut, 1998) over many tasks while the subset of underlying features that are important for the current task can be identified and mapped to actions on a much shorter time-scale i.e. within a single task.

In recent years, Deep RL researchers have shown a lot of interest in attention mechanisms. Most notably, self-attention and the transformer architecture have been applied in Deep RL to push the state-of-the-art further (Manchin et al., 2019, Shen et al., 2019, Zambaldi et al., 2018). At its core, self-attention allows an agent to learn how to attend to different internal representations based on the task at hand by mapping query and key-pair vectors to an output vector (Vaswani et al., 2017). Crucially, this process relies on learning the query, key and value representations via backpropagation in an end-to-end manner. Unfortunately, this reliance on backpropagation for the learning of representations means that self-attention still suffers from the classic criticisms of Deep RL; poor data efficiency and a lack of flexibility, the very issues that attention in biological agents attempts to solve.

**Fig. 1:**
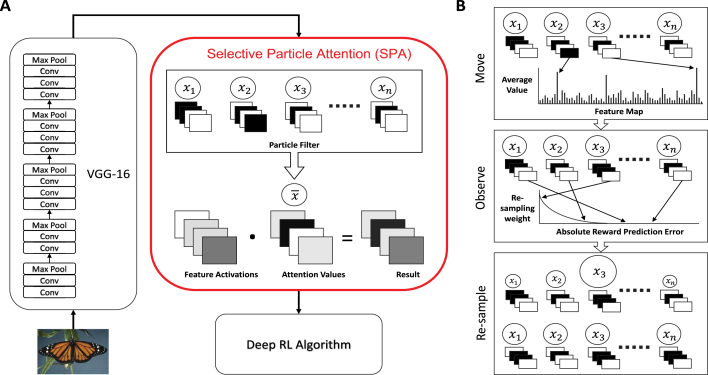
Graphical depiction of Selective Particle Attention (SPA). (A) We use a pre-trained deep convolutional neural network (VGG-16) to extract 2D feature maps from an image. The feature maps are then fed to SPA, which uses a particle filter to generate attention values. Each particle ($x_{i}$) in the particle filter uses a binary vector to represent a hypothesis about which feature maps are useful for the current task. The attention values are calculated as the mean over all particle states, normalised to sum to one ($\overset{\sim }{x}$). Each of the feature maps is multiplied by its corresponding attention value in an element-wise fashion to produce the output of SPA, which is passed to a Deep Reinforcement Learning (RL) algorithm that maps the features to actions using a deep neural network. (B) Updating of the particle filter. In the first (movement) step, a small proportion of particle states ($x_{i}$) are updated to reflect the most active features given the current input. In the second (observe) step, particles are assigned re-sampling weights based on how accurately they predict reward. In the final (re-sample) step, the re-sampling weights are normalised to produce a probability distribution and the particles are re-sampled with replacement.

In order to harness the benefits of selective attention in biological agents we believe that an attention mechanism should be able to rapidly and flexibly select subsets of features without relying on learning new representations via backpropagation. However, such a mechanism faces several key challenges; (i) the number of feature combinations could be extremely large, (ii) reward feedback may be sparse, and (iii) changes in the environment may occur without warning. To address these challenges we present a novel algorithm termed *Selective Particle Attention (*SPA*)* (Fig. 1A), which uses a particle to filter to attend to subsets of features produced by a pre-trained neural network. These features are then passed to a down-stream Deep RL algorithm that maps them to actions using a deep neural network. This leads to a substantial improvement in the data efficiency and flexibility of the Deep RL algorithm. Most importantly, this process does not rely on backpropagation and is therefore able to attend to key subsets of pre-existing features using only reward feedback in a rapid and flexible manner.

We evaluate SPA on two key tasks; a multiple choice task and a 2D video game based on collecting objects. Both tasks involve processing observations from raw pixel input and dealing with unannounced changes in task structure. In both cases the selective attention mechanism of SPA leads to improved performance in terms of the onset, speed, robustness and flexibility of learning compared to approaches that attend to all features, a random subset of features, or employ self-attention. We also show that these findings occur independently of the RL algorithm used, making it applicable to a variety of problems. The remainder of this paper is as divided as follows. In Section 3.2 we provide a detailed description of SPA. In Section 4.1 we assess SPA on a multiple choice task that has a changing reward structure and involves making single decisions. In Section 4.2 we assess SPA on a 2D video game that can change either in reward structure or state space and that requires the agent to make sequential decisions. Finally, in Section 5 we discuss the implications of our findings and highlight promising avenues for future research.

## 2 Related work

### 2.1 Latent representations for Deep Reinforcement Learning

In end-to-end Reinforcement Learning (RL) representations of complex high dimensional inputs, such as pixel values, are learned using a sparse reward signal. This leads to low sample efficiency and representations that are highly specialised for a single task. One way to circumvent these problems is to learn low dimensional representations using other objective functions that have a denser training signal. These representations can then be passed to a Deep RL algorithm, which reduces the complexity of the problem faced by the Deep RL algorithm and leaves the underlying representations unperturbed for future tasks.

In line with this theory, several previous works have used autoencoders to learn low dimensional representations of high dimensional inputs. These low dimensional representations are then used to form the state space of an RL algorithm (Finn et al., 2015, Higgins et al., 2017, Lange and Riedmiller, 2010, Nair et al., 2018, Yarats et al., 2019). Crucially, the authors find that this approach leads to an improvement in the efficiency and robustness of the learned policy compared to a policy learned end-to-end. Similarly, other types of learning such as self-supervised learning can be used to extract features for RL. For example, contrastive learning has been used to learn latent representations for Deep RL algorithms to act upon (Dwibedi et al., 2018, Srinivas et al., 2020). This again substantially improves the efficiency of the Deep RL algorithm.

While applying Deep RL algorithms to latent representations has seen many successes in recent years, we believe this process can be improved further by incorporating a *fast* and *flexible* method for attending to subsets of latent features. By only attending to a subset of features, those not important for the current task can be ignored by the Deep RL algorithm. This further improves efficiency because the dimensionality of the problem is reduced and also further improves flexibility because if the task changes then a new subset of features can be attended to without entirely overwriting the previously learned function.

### 2.2 Attention in Deep Reinforcement Learning

In Deep Reinforcement Learning (RL) attention to subsets of features is typically achieved by multiplying each feature by a value between 0 and 1. A value closer to 1 means that more ‘attention’ is paid to that feature. While this simple filtering process is effective, the difficulty lies in inferring the attention values for a given task. This is particularly difficult when the only feedback is in the form of a sparse, delayed reward signal. The most popular approach for learning a vector of attention values is known as self-attention (Vaswani et al., 2017). Self-attention works by learning three separate representations from the provided input; queries, keys and values. The attention vector is calculated as the dot-product between the queries and keys followed by a softmax function. The output is then the weighted sum of the values, where the weights are provided by the attention vector. This mechanism has been widely used in Deep RL (Bramlage and Cortese, 2021, Iqbal and Sha, 2019, Manchin et al., 2019, Mott et al., 2019, Parisotto et al., 2020, Shen et al., 2019, Wang et al., 2020, Zambaldi et al., 2018) and has been involved in achieving start-of-the-art results (Vinyals et al., 2019).

Despite these successes we believe that self-attention is not an ideal mechanism for selecting subsets of features to be used by a Deep RL algorithm. The primary benefit of self-attention is its ability to relate different parts of the input regardless of how far apart they are. This is particularly useful when trying to solve problems where the input is a sequence (Vaswani et al., 2017) or when abstract relationships within the input need to be represented (Zambaldi et al., 2018). Self-attention’s primary goal is not to identify subsets of features in a rapid and dynamic manner, which is evident from the fact that backpropagation is used to learn the query, key and value representations. This reliance on backpropagation means that the underlying attention mechanism is slow to train and inflexible; these are the exact issues that attending to subsets of features should help to alleviate.

Attempts to learn an attention vector without backpropagation have been limited. Most notably, Yuezhang, Zhang, and Ballard (2018) explored hand-crafting attention values or inferring them with optical flow. Hand-crafting attention values is effective but circumvents the problem of learning the values, which is the focus of this work. In comparison, optical flow can only be applied to problems involving visual movement and is fixed regardless of reward structure. This means that it captures bottom-up attention, where attention is influenced by the inherent properties of the input, but not top-down attention, where attention is guided by current goals and reward structure.

### 2.3 Particle filters

Filtering-based approaches have been widely used to deal with uncertainty and non-linear dynamics in Markov processes (Rudenko, 2019, Zhang, He et al., 2021, Zhang, Wang et al., 2021). In the present work we propose that particle filters in particular can be used to rapidly and dynamically learn which features to attend to by combining both bottom-up and top-down attention. Particle filters rely on approximate inference and importance sampling to infer the values of a hidden variable given sequential observations (Doucet, Johansen, et al., 2009). As a result, they have been used in Deep Reinforcement Learning (RL) to deal with the problem of partially observable Markov decision processes (POMDPs), where the true Markovian state of the environment is unknown and must be inferred from past observations (Besse, 2009, Igl et al., 2018, Ma et al., 2020).

In the present work we suggest that particle filters have several properties that also make them a good candidate for rapidly and dynamically attending to subsets of pre-learned features. Firstly, they use approximate inference, which is important because the number of feature combinations to consider can be prohibitively large. Secondly, particle filters are able to deal with non-linear dynamics and non-gaussian distributions, which are typical of RL problems. Finally, and most importantly, particle filters rely on sequential importance sampling rather than backpropagation. This means that they can be updated rapidly and dynamically in comparison to approaches such as self-attention.

## 3 Model implementation

### 3.1 Bio-inspired features of Selective Particle Attention (SPA)

Our approach is heavily inspired by selective attention in biological agents. Selective attention is the process of selecting subsets of pre-existing features for decision-making and is thought to be the responsibility of the Pre-Frontal Cortex (PFC) (Bichot et al., 2015, Desimone and Duncan, 1995, Miller and Cohen, 2001, Paneri and Gregoriou, 2017). For example, Selective Particle Attention (SPA) applies attention by multiplying each feature by a value between 0 and 1, which mimics the heightening and dampening of responses often associated with biological attention (Saenz et al., 2002, Treue and Trujillo, 1999). These values are learned via a particle filter, which has been proposed to be a plausible computational model of biological attention (Radulescu, Niv and Ballard, 2019) that can model shifts in attention better than gradual error-driven learning (Radulescu, Niv and Daw, 2019). Each particle of the filter is used to represent a hypothesis about which features are important for the current task. This reflects the way the PFC represents competing hypotheses about which features of the visual input to attend to given the current goal (Miller and Cohen, 2001, Radulescu, Niv and Ballard, 2019). It also consistent with Bayesian theories of the brain that suggest flexible behaviour is based on approximate inference and hypothesis testing (Donoso et al., 2014, Jiang et al., 2014, Koechlin, 2014).

The states of each particle are updated based on (1) how accurately the combination of features predicts future reward and (2) the magnitude of activation of individual features. The first step reflects top-down attention and is based on a theoretical proposal from neuroscience that humans learn to attend to the features that are most predictive of reward (Mackintosh, 1975). For example, people are able to switch between an object-based or a feature-based state representation based on which one is the best predictor of reward (Farashahi, Rowe, Aslami, Lee, & Soltani, 2017). The second step reflects bottom-up attention and is inspired by the fact that individual features with inherent saliency play an important role in the biasing of biological attention (Desimone and Duncan, 1995, Treue, 2003). Once the particle states have been updated via these two steps, the attention values are calculated by averaging the particle states and normalising the result to sum to one. This is in line with computational neuroscience models of visual attention, which highlight normalisation as a key step for capturing competition between features in the visual cortex (Reynolds & Heeger, 2009).

### 3.2 Algorithmic details

#### 3.2.1 Attention vector

In order to filter the pre-learned representations for the Deep RL algorithm, Selective Particle Attention (SPA) uses an *attention vector* to perform soft-attention. The attention vector has K dimensions, where each entry can take on any real valued number between 0 and 1 inclusive:

(1) $$A\in {\left[0,1\right]}^{K}$$

where K is the number of pre-learned features, which in our case was 512. This attention vector is applied to the pre-learned features using element-wise multiplication between A and the values of each feature. In the case of VGG-16, each entry in A is replicated to match the dimensions of a single feature map, with the same attentional value applied across all spatial locations. This process re-weights the feature map activations, amplifying feature maps with a large attentional weight while maintaining all spatial information. The output is then reshaped into a single vector and passed on to a Deep Reinforcement Learning (RL) algorithm that maps it to a chosen action.

#### 3.2.2 Particle filter

Rapidly and flexibly learning the values of the attention vector in an RL setting is difficult because feedback is a sparse scalar reward and actions have temporal consequences. To solve this problem, we use a particle filter to learn the values of the attention vector given the current task. Particle filters are typically used to estimate the value of a latent variable (X) given noisy samples of an observed variable (O) when the number of potential values is large. The overall goal of a particle filter is to use a set of ‘particles’ to represent a posterior distribution over the latent variable. Each particle represents a belief or hypothesis about the value of the latent variable and the density of the particles can be used to approximate the posterior distribution.

In our case the latent variable X represents the configuration of features that are useful for the current task. We use a value of 1 to denote a feature as useful and a value of 0 to denote a feature as not useful. This means that x can be any binary vector of size K:

(2) $$x\in {\left\{0,1\right\}}^{K}$$

where K is the number of features, which in our case was 512 corresponding to the number of feature maps of VGG-16. As the number of possible values that X can take is $2^{K}$, we use N particles to approximate the posterior distribution over X where $N\ll 2^{K}$. The state $x^{i}$ of the ith particle therefore corresponds to a binary vector of length K and provides a hypothesis about which features it deems relevant for the current task.

A particle filter consists of two main steps; a movement step and an observation step. In the movement step, the particles are updated based on some known transition probability for the latent variable:

(3) $$x^{\prime }∼P\left(X^{\prime }|x\right)$$

This process is often used to represent the passing of time. In our approach we introduce the notion of bottom-up attention during the movement step. Let ${\bar{f}}_{t}^{k}$ denote the average value over all the units in feature map k from VGG-16 at time t. At each time-step t a particle is updated as follows with some probability ϕ:

(4) $$v_{t}^{k}=\frac{{\bar{f}}_{t}^{k}}{\overset{K}{\underset{j=1}{\sum }}{\bar{f}}_{t}^{j}}$$

(5) $$p^{k}=\frac{\exp\left(v_{t}^{k}*\tau_{\text{BU}}\right)}{\underset{j}{\max}\exp\left(v_{t}^{j}*\tau_{\text{BU}}\right)}$$

(6) $$P\left(x_{k}^{\prime }=n\right)=\left\{\begin{array}{cc} p^{k}\; & \text{for }n=1\\ 1-p^{k}\; & \text{for }n=0 \end{array}\right)$$

First the mean activation values for each feature map of VGG-16 are normalised to sum to 1. This normalisation accounts for differences in overall activation values between time steps and preserves relative differences between activation values. The activation values are then exponentiated and normalised by the maximum value across all feature maps. This ensures that the most active feature will receive a value of 1. Finally this is used as the probability that the kth entry of the particle state will be equal to one, as described by a Bernoulli distribution. In this way, a proportion of the particles are updated to represent the most active features given the current input. This is akin to bottom-up attention, whereby highly salient perceptual features capture one’s attention in an involuntary manner. In our agent this serves to introduce a prior to attend to the highly active features of the current task. The free parameter $\tau_{\text{BU}}$ controls the strength of the bottom up attention, a higher value of $\tau_{\text{BU}}$ leads to a higher probability of the most active features being attended to and the least active features not being attended to.

In the observation step of a particle filter, particles are weighted based on the likelihood of the observed variable O given the value of the latent variable X represented by a particle. These weights are then used to re-sample the particles and update the posterior distribution ready for the next time step. In our approach, we introduce top-down attention during this step. We take our observed variable O to be the return from a given state $R_{t}$. We calculate the likelihood of this return by using the particle state as the attention vector and calculating the error between the predicted state value and the return. Let $x^{i}$ denote the state of the ith particle, the likelihood for $x^{i}$ is calculated as follows:

(7) $$A_{k}^{i}=\frac{x_{k}^{i}}{\overset{K}{\underset{j=1}{\sum }}x_{j}^{i}}$$

(8) $$\delta^{i}={\left(R_{t}-V\left(s_{t};A^{i}\right)\right)}^{2}$$

(9) $$P\left(R_{t}|x^{i}\right)\propto \exp\left(-\left(\delta^{i}-\underset{j}{\min}\delta^{j}\right)*\tau_{TD}\right)$$

where $R_{t}$ is the return from state $s_{t}$ and $V\left(s_{t};A^{i}\right)$ is the predicted state value calculated by using the normalised particle state $x^{i}$ as the attention vector. The normalisation step in Eq. (7) accounts for the different numbers of features that are attended to by different particles. Eq. (8) calculates the squared error between the return and predicted state value. The likelihood of the return $R_{t}$ is then proportional to this error value. This process can be seen as evaluating the accuracy of a particle’s hypothesis over which features are relevant for the given task. If the particle’s hypothesis is good then it will more accurately predict the target return and so will produce a larger likelihood. In this way we capture the effect of top-down attention, whereby a set of hypotheses are evaluated and the most accurate ones are considered for the next time step. $\tau_{TD}$ controls the strength of this top down attention; a larger value will more strongly penalise hypotheses that are inaccurate.

**Table 1:** Hyper-parameter values used for the Multiple Choice task (MC) and Object Collection game (OC).

Parameter	Value (MC)	Value (OC)	Description
N	250	250	Number of particles
$\tau_{BU}$	10	1	Strength of bottom-up attention
$\tau_{TD}$	10	10	Strength of top-down attention
$c_{max}$	1	1000	Frequency of attention updates
$t_{max}$	1	10	Maximum length of return
ε	.2	–	Probability of selecting a random action
λ	.00025	–	Learning rate for RMSProp
κ	.95	–	Momentum for RMSProp
ι	.01	–	Constant for denominator in RMSProp
β	–	0.01	Exploration strength
γ	–	.99	Discount factor for future rewards
m	–	8	Number of frames skipped
α	–	0.0001	Learning rate for Adam optimiser
d	–	128	Dimension of the query and key vectors for self-attention

Once the likelihoods have been calculated they are normalised to form a probability distribution and the particles are re-sampled with replacement:

(10) $$P\left(x^{\prime }\right)=\frac{\overset{N}{\underset{i=1}{\sum }}P\left(R_{t}|x^{\prime }\right)I\left(x^{i}=x^{\prime }\right)}{\overset{N}{\underset{i=1}{\sum }}P\left(R_{t}|x^{i}\right)}$$

(11) $$x^{\prime }∼P\left(x^{\prime }\right)$$

Once the re-sampling has been performed, the final step is to reset the value of the attention vector. This is done by setting the attention vector to be the mean of the particle states and then normalising the vector to sum to one:

(12) $${\bar{x}}_{k}=\frac{1}{N}\overset{N}{\underset{n=1}{\sum }}x_{k}^{n}$$

(13) $$A_{k}=\frac{{\bar{x}}_{k}}{\overset{K}{\underset{j=1}{\sum }}{\bar{x}}_{j}}$$

where N is the number of particles and K is the number of features. The full algorithm used to update the attention vector can be seen in Algorithm 1 in the Appendix.

### 3.3 Modular design choices

In order to assess how well Selective Particle Attention (SPA) can rapidly and flexibly attend to subsets of pre-learned features, we have to decide on a method for extracting the features and on a Deep RL algorithm for learning a control policy from these features. A vast array of approaches exist for feature extraction that are compatible with SPA. We chose to use the pre-trained Deep Convolutional Neural Network (DCNN) VGG-16 (Simonyan & Zisserman, 2014) because it can be applied to raw pixel values, it is widespread in the machine learning literature and it has a good correspondence with representations found in the visual stream of biological agents (Schrimpf et al., 2018). Similarly, there are many Deep RL algorithms that can be applied to pre-learned features. Often this decision is influenced by the nature of the problem you are trying to solve, for example whether it involves a continuous or discrete action space. In our case the only constraint is that the Deep RL algorithm must estimate a value function. This is critical because it allows SPA to decide whether features should be attended to or not based on how well they predict future reward (see Section 3.2). For the first experiment (multiple choice), we used a Deep Neural Network to estimate the value of each image and chose the image with the highest value in an ε-greedy manner. The network was trained to minimise the difference between the predicted value of the chosen image and the actual reward obtained, which is equivalent to performing a one-step Monte Carlo backup (Sutton & Barto, 1998). For the second experiment (object collector), we chose to use Advantage Actor–Critic (A2C) (Mnih et al., 2016), which has been widely used for solving video games.

More details of these algorithms can be found in the Appendix and all of the hyper-parameter values are shown in (Table 1). The majority of these values are standard for Deep RL based on the types of problems that we are exploring. However, the number of particles, the frequency of attention updates, and the strength of the bottom-up and top-down attention are unique to our approach (SPA). We therefore conducted an informal parameter search over these parameters and found that the results were largely consistent. We chose the final values based on their order of magnitude.

## 4 Experiments

### 4.1 Making single-step decisions in the face of a changing environment

**Fig. 2:**
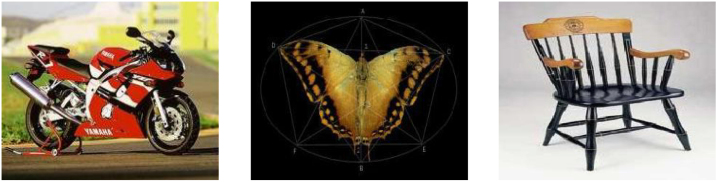
Example images from the Caltech 101 data set, which consists of 101 object categories with approximately 40 to 800 images per category. Left image is from the ‘motorbikes’ category, the middle image is from the ‘butterfly’ category and the right image is from the ‘chair’ category. On each trial the agent was presented with three separate images each selected randomly from three different categories. For a given block, only one image category was associated with a reward of ＋1 and the rest were associated with a reward of 0. The rewarded category was chosen at random for each block of trials.

In order to test the effectiveness of SPA we designed two tasks that require an agent to select actions based on raw pixel inputs and to switch attention without warning in response to changing task demands. In both tasks the score used to evaluate the agent was the achieved reward because this was the value the algorithm was tasked with optimising. The first task consisted of multiple choice questions using images from the entire Caltech 101 data set (Fei-Fei, Fergus, & Perona, 2006), which consists of 101 object categories with approximately 40 to 800 images per category. At the start of the task, three object categories were randomly chosen from the Caltech 101 data set and one of them was randomly designated as the ‘target’ category. Each question then consisted of presenting one image randomly sampled from each of the three categories (Fig. 2). The image from the ‘target’ category was associated with a positive reward of ＋1 while the images from the other categories were associated with no reward. Crucially, after every 50 questions, the ‘target’ category was changed at random.

The purpose for this task design was two-fold. Firstly, the agent has to work out on each trial which image is associated with a reward based on the features of the ‘target’ category. For example, if the target category is ‘soccer ball’ then the agent has to learn to attend to features that are indicative of round shapes in order to obtain reward. Secondly, because the ‘target’ category changes every 50 trials, the agent also has to adapt to this change in reward structure by dynamically shifting its attention to the features of another category using only reward feedback.

**Fig. 3:**
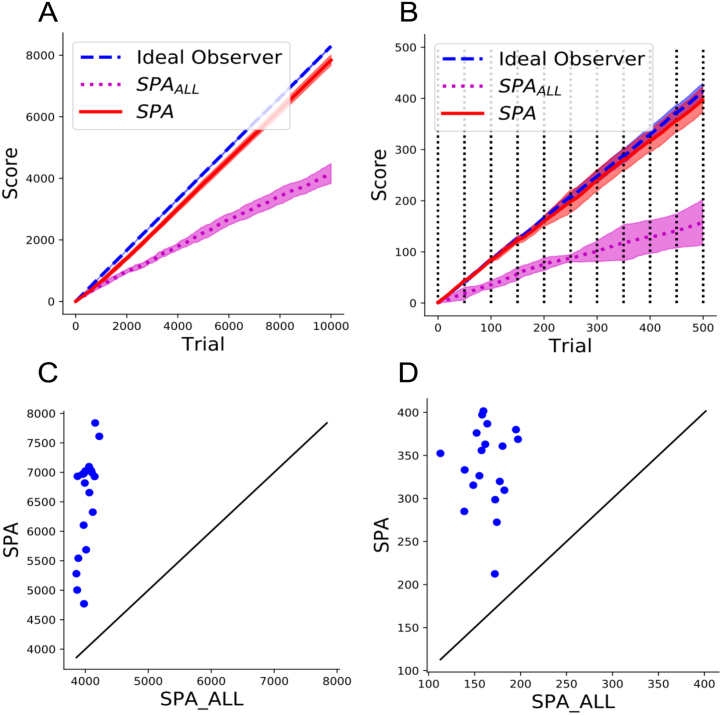
Performance of Selective Particle Attention (SPA) on the multiple choice task. (A–B) Training (A) and test (B) reward over 5 random seeds for the leopards, faces and soccer balls image categories. The rewarded image category was changed randomly every 50 trials and is represented by a vertical black line in B. Error bars represent one standard deviation. SPA used selective attention to attend to features that it deemed useful for the current task. $SPA_{ALL}$ did not use selective attention and instead attended to every feature of VGG-16. The Ideal observer model represents ceiling performance. (C–D) Comparison of SPA and $SPA_{ALL}$ in terms of total training (C) and test (D) reward for 20 different image category combinations. Each point represents the mean performance over 5 random seeds for an image category combination. Solid line represents equal performance between SPA and $SPA_{ALL}$.

To ensure that the agent did not learn to remember specific exemplars in the data set we had training and test phases. During the training phase the agent was allowed to update the parameters of the Deep RL network in response to reward feedback but during the test phase it was not. Therefore the only parameters the agent was allowed to change during the test phase was the attention values using the particle filter. Importantly, the test phase used images from the chosen object categories that were not presented during training. In total, the training phase consisted of 200 blocks of 50 trials and the test phase consisted of 10 blocks of 50 trials.

As a baseline to measure the effectiveness of our proposed attention mechanism we compared the performance of SPA to a version of SPA where each entry of the attention vector was set to a fixed value of 1/K. This corresponds to attending to all features of VGG-16 equally and we refer to this approach as $SPA_{ALL}$. We also ran an ideal observer model on the multiple choice task to provide a measure of ceiling performance. The ideal observer model selects the image corresponding to the last rewarded object category. All models select a random action with probability ε in order to encourage exploration.

Fig. 3A shows the performance of SPA, $SPA_{ALL}$ and the ideal observer during training on the multiple choice task for one random combination of object categories over 5 random seeds. In this example SPA performs close to optimal as it shows a similar learning trajectory to the ideal observer. In comparison, $SPA_{ALL}$ performs poorly and is substantially worse than both SPA and the ideal observer. To test the robustness of these findings we ran all three approaches over 5 random seeds on 20 different combinations of object categories. Fig. 3C shows the results of these simulations. SPA out-performed $SPA_{ALL}$ during training for every combination of categories that we tested.

While the dynamic attention mechanism of SPA appeared to provide a substantial benefit during training, we also wanted to test whether this benefit generalised to unseen images. Fig. 3B shows the results of the three approaches on the test phase after the training seen in Fig. 3A. Again the rewarded image category was changed every 50 trials. Importantly the test blocks used images that were not used during training and all the weights of the Deep RL algorithm were frozen so that only the attention vector could change in the case of SPA. Again SPA exhibited performance similar to that of the ideal observer, while $SPA_{ALL}$ showed significantly worse performance. Fig. 3D shows the test results over 5 random seeds for all 20 of the different category combinations. As with training, SPA out-performed $SPA_{ALL}$ for all of the category combinations. These results suggest that the benefit of the attention mechanism of SPA generalises well to unseen images.

**Fig. 4:**
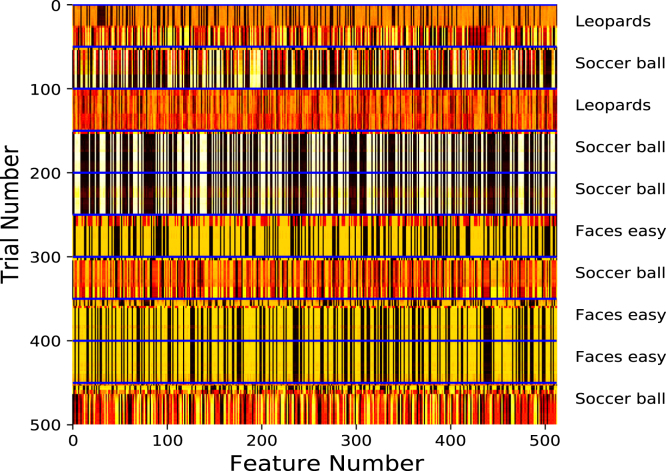
Example of the attention vector values for the leopards, faces and soccer balls image categories. Black represents a value of 0 and white represents a value of 1. The solid blue horizontal line represents a random change in the rewarded image category. The rewarded image category for a given block is presented on the right.

Both the training and test results suggest that SPA is able to cope with changes in the reward function by dynamically re-configuring existing representations for the purpose of state evaluation. Fig. 4 shows an example of the attention vector during the test phase. The attention vector reliably changes when the target image category changes. This confirms that the attention mechanism of SPA is able to use changes in the reward function to re-evaluate the features that need to be attended to. Interestingly, the attention vector is not the same every time a given image category is made the target. This is likely due to the fact that SPA will be biased towards attending to features that are present in the first few images, which will be different for each block. In addition, there is likely to be a contextual effect of the rewarded image category in the previous block. For example, if in the previous block the category ‘soccer ball’ was rewarded and in the current block ‘faces’ are rewarded, then this might bias the selection of features that correspond to ‘round’ in the current block.

**Fig. 5:**
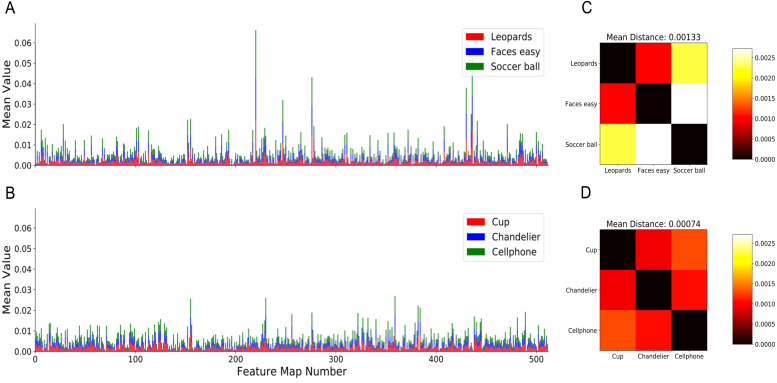
Analysis of the feature map values produced by VGG-16 during the multiple choice task. (A–B) Average feature map values of VGG-16 for the combination of image categories that lead to the best (A) and worst (B) training performance of SPA. (C–D) Euclidean distances between the average feature map values of VGG-16 for the combination of image categories that lead to the best (C) and worst (D) training performance of SPA.

The results shown in Fig. 3B and D indicate that the performance of SPA can vary depending on the combination of image categories that are chosen. This suggests that SPA finds it easier to discriminate between certain image categories compared to others based on their features. Fig. 5A and B show the mean feature map values of VGG-16 for the image categories that SPA performed best (leopards, faces and soccer balls) and worst (cups, chandeliers and cellphones) on. In the best case scenario, the feature maps contain several features that are substantially more active than others. This likely provides a good substrate for bottom-up attention because there are a handful of features that are reliably more active than the others, which corresponds to a strong prior over hypotheses. In comparison, in the worse case scenario, the features take on a more uniform distribution of activation values and so the prior over hypotheses is weaker.

Bottom-up attention aside, having a few highly active features is only useful for the multiple choice task if they help to discriminate between the different image categories. As seen in Fig. 5A and B, each image category produces 512 average feature values, which can then be expressed as a vector in Euclidean space. Fig. 5C and D show the Euclidean distance between these vectors for the best and worst case scenarios respectively. In the best case scenario, the Euclidean distance between the majority of the image categories is larger than in the worst case scenario. In addition the mean Euclidean distance over all pairwise comparisons in the best case scenario is nearly double that of the worst case scenario. This suggests that the categories in the best case scenario are easier to discriminate between because they are further apart in Euclidean space. This is likely to help the top-down attention of SPA because each particle will produce very different value estimates depending on the image category being attended to.

### 4.2 Making multi-step decisions in the face of a changing environment

Having demonstrated the effectiveness of SPA in the multiple choice task, we next wanted to investigate whether this translated to more complex tasks involving sequential decision-making. To this end we designed a 2D video game, which we refer to as the object collection game, using Pygame (www.pygame.org). In the game, the agent controlled a grey block at the bottom of the screen and could move it either left or right at each time step. An object, which could vary in shape and colour, was generated every second at the top of the screen and moved downwards to the bottom of the screen in a straight line. The goal of the agent was to collect objects by colliding with them as they reached the bottom of the screen. Importantly, the agent was only rewarded for collecting objects that had a specific colour and/ or shape, which encourages the agent to attend to specific features that are indicative of reward. Agents were trained for 2000 episodes, with each episode lasting 60 s.

**Fig. 6:**
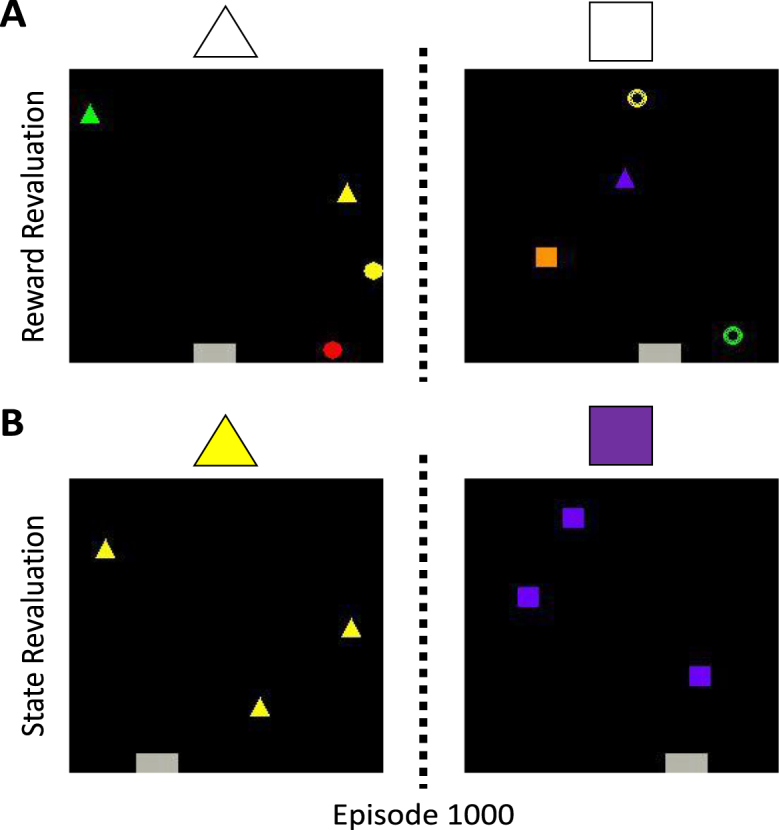
Example screenshots from the two variations of the object collection game. The agent is in control of the grey rectangle and can move it either left or right to catch objects that move from the top of the screen to the bottom. The rewarded object is depicted above each screenshot. (A) The reward revaluation task. A specific shape is associated with a reward of ＋1 regardless of its colour. All other shapes are associated with a reward of 0. The rewarded shape is then randomly changed after 1000 episodes, which corresponds to a change in the reward function of the environment. (B) The state revaluation task. All objects are the same shape and colour, and are associated with a reward of +1. The shape and colour of these objects is then randomly changed after 1000 episodes, which corresponds to a change in the state space of the environment.

We explored two different variations of this basic game configuration. In the first variation, the agent was given a reward of ＋1 for collecting objects of a certain shape and a reward of 0 for all other shapes (Fig. 6A). This tested the agent’s ability to focus on particular features of the environment and ignore others; the agent had to attend to the target shape while ignoring colour and all other shapes. As we saw in the multiple choice task, one benefit of an effective selective attention mechanism is that attention can be altered in response to changes in the reward structure. To test whether this was also the case in the object collection game, we randomly selected a new shape to be rewarded after 1000 episodes. This is akin to changing the reward function of the environment and requires the agent to re-evaluate its policy. We refer to this variation as the reward revaluation task.

In addition to the reward function, other aspects of the environment can change such as the set of perceptual observations experienced by the agent. In RL this is commonly referred to as a change in state space. The second variation of the object collection game therefore explored the ability of SPA to deal with changes in state space (Fig. 6B). For the first 1000 episodes, all the objects were the same shape and colour and were associated with a reward of +1. After 1000 episodes the shape and colour of the objects were changed to a different shape and colour. This corresponds to a change in the perceptual input of the agent because the objects present in the first half of training are different to the ones present in the second half of training. We refer to this variation as the state revaluation task.

As with the multiple choice task, we compared SPA to a baseline approach that attended to all features of VGG-16, which we refer to as $SPA_{ALL}$. In addition to $SPA_{ALL}$, we also included two other conditions for comparison. The first condition set the attention vector to a random binary vector, which was then normalised to sum to 1. This condition was included to account for the fact that random feature reduction may lead to improved performance and we refer to it as $SPA_{RANDOM}$. The second conditioned used self-attention rather than SPA to attend to the features of VGG-16. We chose to compare SPA to self-attention because it is a standard state-of-the-art attention mechanism in Deep RL and relies upon backpropagation rather than approximate inference. Self-attention was implemented as follows; the features from VGG-16 were used to learn a query vector and K key vectors, where K is the number of feature maps of VGG-16. The standard self attention update (Vaswani et al., 2017) was then applied to obtain an attention vector with K dimensions that summed to one, which mirrors the attention vector of SPA. The attention vector was then applied to the features of VGG-16 in the same way as SPA.

**Fig. 7:**
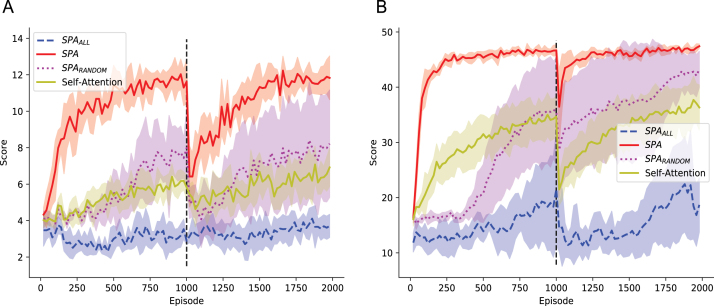
Performance of SPA, $SPA_{ALL}$, $SPA_{RANDOM}$ and self-attention on the object collection game. Each point is the average reward over the last 20 episodes. Error bars represent one standard deviation. Each approach was run over 5 different random seeds and the error bars represent one standard deviation. SPA used selective attention to attend to features that it deemed useful for the current task. $SPA_{ALL}$ did not use selective attention and instead attended to every feature of VGG-16. $SPA_{RANDOM}$ attended to a random subset of features, which changed with each random seed (A) The vertical dashed line indicates where the rewarded object was changed without warning. (B) All objects were the same colour and shape. The vertical dashed line indicates where the shape and colour of these objects was changed without warning.

Fig. 7A shows the results of SPA, $SPA_{ALL}$, $SPA_{RANDOM}$ and self-attention on the reward revaluation task. Focusing on the first 1000 episodes before the change in reward function, SPA significantly out-performed $SPA_{ALL}$, $SPA_{RANDOM}$ and self-attention over the course of learning. $SPA_{ALL}$ saw the worst performance showing little evidence of learning over the first 1000 episodes. This suggests that naively learning over all features is a highly ineffective strategy given a limited amount of experience. In contrast, $SPA_{RANDOM}$ and self-attention showed evidence of gradual learning after around 500 episodes. The learning of $SPA_{RANDOM}$ was highly variable as would be expected given that the features were randomly selected for on each random seed. Nevertheless this demonstrates that simply reducing the number of features at random is sufficient to provide a learning benefit. SPA began improving its reward almost immediately after a handful of episodes. This onset of learning is noticeably earlier than the other approaches. Not only did learning occur earlier but it was also much faster, as indicated by the sharper increase in reward compared to the other approaches. As a result, by episode 1000 SPA was able to achieve a higher reward than the other approaches. Importantly, SPA dramatically out-performed self-attention highlighting the benefit of using approximate inference for attention as opposed to backpropagation. Overall, these results suggest that the particle filter mechanism of SPA is able to identify features useful for value computation and that this translates into a substantial learning benefit given the current task.

Upon the change in reward function after 1000 episodes, $SPA_{ALL}$ continued to demonstrate no evidence of learning. In comparison, $SPA_{RANDOM}$ and self-attention showed a decrease in performance as soon as the reward function changed and they were slow to recover performance. SPA also showed a marked decrease in performance immediately after the change in reward function. This is to be expected as the agent had no prior warning that the reward function was about to change. However, the performance of SPA dropped to a level that was still above $SPA_{RANDOM}$ and self-attention. The performance then remained significantly higher than $SPA_{RANDOM}$ and self-attention throughout the recovery in performance. This resulted in SPA achieving a higher reward than all other approaches on the final episode. These results suggest that the particle filter mechanism of SPA is better equipped to handle changes in the environment by quickly re-evaluating which features are important for the current task.

Fig. 7B shows the results of the four approaches on the change in state space task. As before, for the first 1000 episodes SPA demonstrated evidence of learning that occurred earlier, faster and more robustly than the other approaches. Upon the change in state space after 1000 episodes, $SPA_{ALL}$ dropped back to starting levels of performance and was slow to recover to a low level of performance. In comparison, SPA showed a slight decrease in performance but was quickly able to recover to a high level of performance. Both $SPA_{RANDOM}$ and self-attention also saw a drop in performance but the rate of recovery was slower than SPA and only reached an intermediate level of performance. Interestingly, self-attention saw a larger drop in performance than $SPA_{RANDOM}$. This highlights one of the key issues of using self-attention and backpropagation for an attention mechanism; the query and key representations are learned specifically for the first task and have to be slowly re-learned when the task changes. In comparison, the fixed attention of $SPA_{RANDOM}$ may already include some features that are useful for the second task and so it is less affected by the change in task. These results further support the idea that the selective attention mechanism of SPA is able to use approximate inference to quickly adapt to changes in the current task by re-orientating its attention towards a different set of features. In addition, the consistently high performance of SPA and its fast rate of recovery during the change in state space indicates that much of the knowledge learned by the Deep RL algorithm in the first 1000 episodes was still of use in the second 100 episodes. This suggests that the selective attention mechanism of SPA is also able to promote the transfer of knowledge between tasks.

## 5 Discussion

Learning latent representations for Deep Reinforcement Learning (RL) can lead to many benefits including a lower dimensional input, access to a denser training signal and representations that are useful for multiple tasks. Typically, when latent or pre-learned representations are used for Deep RL all of the extracted features are used to learn a policy. However this naive approach can be improved upon by only passing the features that are relevant for the current task to the Deep RL algorithm. This improves learning efficiency because the dimensionality of the input is reduced further and also improves flexibility because there is less interference between features when the task changes. Despite these benefits, mechanisms for attending to subsets of features in Deep RL tend to rely on backpropagation which makes them slow to learn and inflexible to changes in task. As a result, this negates the many potential benefits of being able to rapidly and dynamically attend to subsets of features. To solve this problem we proposed a novel algorithm, termed Selective Particle Attention (SPA), that uses a particle filter to attend to subsets of features using only reward feedback, and sequential importance sampling instead of backpropagation.

### 5.1 Key results

We evaluated SPA on two different tasks. The first task was a multiple choice task involving naturalistic images. SPA was able to achieve close to ceiling performance on this task for several examples and dramatically out-performed a naive version that attended to all features. Inspection of SPA’s vector of attention values showed that it was able to quickly change its configuration in response to changes in the reward function. This highlights how SPA can be used to quickly respond to unannounced changes in the environment and improve the overall efficiency of learning.

The aforementioned multiple choice task can be viewed as a single step Markov Decision Process (MDP) or a supervised learning problem where the network has to predict the reward associated with each image. SPA can therefore theoretically be applied to a range of classification or regression problems such as MNIST (Deng, 2012) or CIFAR-10 (Krizhevsky, Hinton, et al., 2009). However, the purpose of this research was to explore whether SPA could be applied to RL tasks/multi-step MDPs. This is a substantially harder problem because there are temporal dependencies and the reward feedback is both delayed and sparse. With this in mind, we chose the second task to be a simple 2D video game. As with the multiple choice task, SPA was able to perform significantly better than the naive approach that attended to all features. This provides evidence that SPA can improve the efficiency of learning on complex RL problems that require temporal dependencies. It also demonstrates that SPA is a general approach that can be used with a variety of Deep RL algorithms so long as they estimate a value function. In addition to out-performing the naive approach, SPA also performed better than an approach that randomly selected a subset of features to attend to. This demonstrates that SPA is able to attend to features in a targeted manner and that the benefits of SPA are not simply due to random dimensionality reduction. As a final baseline on the 2D video game, we compared SPA to self-attention and again SPA performed significantly better. This highlights the benefit of using approximate inference instead of backpropagation to rapidly and dynamically attend to features.

Interestingly, in order to successfully apply SPA to the 2D object collection game the attention vector was only updated every 1000 time-steps rather than on every time step. This was necessary because it allowed the Deep RL algorithm time to respond to the change in attention and attempt to learn a useful function of the currently attended features. This is akin to a person learning based on a single hypothesis for a fixed amount of time before deciding whether to change to another hypothesis or not. This may explain why attentional inertia is prevalent in children and adults (Anderson et al., 1987, Burns and Anderson, 1993, Longman et al., 2014, Richards and Anderson, 2004), as the brain requires time to evaluate a given hypothesis before deciding whether to switch attention. Future work should systematically explore this apparent trade-off between the potential benefits of switching to a new hypothesis and the time needed to sufficiently evaluate a hypothesis.

Part of the reason for the design of the object collection game was that it allowed for the rewarded shape to be easily changed during learning. This change can be manipulated to correspond to a change in the reward function or state space. We found that SPA was significantly better equipped to deal with such changes compared to other naive approaches that either attended to all or a random subset of features. In particular, SPA showed an extremely fast recovery in response to a change in state space. This is often considered an extremely difficult problem as deep neural networks typically fail catastrophically when the input distribution is changed (Lake et al., 2017). Nevertheless, the small drop in performance and rapid recovery to asymptotic levels suggests that SPA was able to transfer much of the knowledge that it had acquired before the change in state space. These results further support the findings of the multiple choice task, which demonstrated SPA’s improved ability to deal with changes in the environment.

### 5.2 Why selective particle attention is beneficial

We believe the ability of SPA to focus on a subset of features based on the current task is beneficial for several reasons. Firstly, it helps to reduce the dimensionality of the problem, which reduces the impact of noisy features and the complexity of the function that needs to be learned. This improves generalisation because the learned function does not fit spurious features and therefore ignores any changes to them. This benefit of dimensionality reduction can even be seen in the case of $SPA_{RANDOM}$, which out-performed $SPA_{ALL}$ on the object collection game. SPA takes this one step further however, by providing targeted dimensionality reduction rather than selecting a random subset of features. Furthermore, the selective attention of SPA helps to reduce interference between tasks by guiding learning onto different sets of features. This results in the learning of different sub-networks that can be quickly identified based on the current task. This greatly improves the capacity of the Deep RL algorithm to represent different functions and to switch between them in the face of changes to the environment. Finally SPA uses approximate Bayesian inference, rather than backpropagation, to learn which features to attend to and this leads to learning on a much faster time scale.

### 5.3 The mechanisms behind selective particle attention

The key component of SPA is a particle filter, which is used to infer the vector of attention values using only reward feedback. One of the primary benefits of using a particle filter is that it relies on sampling to produce an approximate value of a hidden variable. This is important because the potential number of feature combinations that need to be evaluated for a given task can be extremely large. In our case there were $2^{512}$ potential feature combinations and so it would be computationally infeasible to evaluate all of them. However, by using only 250 particles we were still able to converge to a satisfactory solution thanks to the iterative re-sampling procedure of the particle filter. Future work should explore how the number of particles in SPA affects its ability to find the best combination of features and therefore its asymptotic performance on tasks. The number of particles may be a limitation in real world applications where inference time is critical and computational resources are limited. If the number of underlying features is large then many particles may be required to approximate the posterior distribution. However, it is worth noting that, as the particles converge, the likelihood of the observed variable need only be calculated once for particles with identical states.

The movement step of the particle filter is guided by bottom-up attention, which introduces a bias towards the most active features. This is equivalent to a prior over the hypothesis space, favouring hypotheses with relatively few features. Interestingly, this is consistent with findings in neuroscience that people tend to make decisions based on individual features before reasoning about objects that involve more complex combinations of features (Choung et al., 2017, Farashahi et al., 2017). Similarly, people find it harder to perform classification tasks as the number of relevant dimensions increases (Shepard, Hovland, & Jenkins, 1961). The bottom-up attention captured in the movement step of the particle filter therefore seems to introduce a biologically plausible bias over the hypothesis space.

The observation step of the particle filter is guided by top-down attention. This process involves evaluating different hypotheses based on their ability to predict future reward (Mackintosh, 1975). It has been proposed that a similar mechanism may occur in the corticostriatal circuitry of humans (Radulescu, Niv and Ballard, 2019). The proposal states that different pools of neurons in the PFC may represent different hypotheses about the structure of the current RL task. These pools then compete via lateral inhibition and connections to the striatum help to favour pools that lead to reward. This process parallels the observation step of SPA, whereby hypotheses that are more predictive of reward are more likely to be re-sampled and out-compete other hypotheses. In addition, the phenomenon of representing and testing multiple hypotheses during RL appears to be prevalent in human populations (Wilson & Niv, 2012).

### 5.4 Dependency on underlying representations

The effectiveness of SPA naturally depends on the nature of the features or representations that it is attending to. If in real world applications the underlying representations are not abstract enough and there is not a subset of features that are sufficient to achieve the desired task performance then the effectiveness of SPA will be limited. In the human brain it has been proposed that the usefulness of selective visual feature attention decreases as you move back through the visual stream (Lindsay & Miller, 2018). This is because features present later in the visual stream consist of higher-order representations that are increasingly abstract. For example, one of the object categories in the multiple choice task was faces, which are known to be represented later on in the visual stream of the brain (Grill-Spector, Weiner, Gomez, Stigliani, & Natu, 2018). Having such a representation makes the multiple choice task easy for the brain because it only has to attend to one feature rather than a collection of low level features. This also reduces the need to consider lots of complicated hypotheses. Future work should test whether SPA displays similar behaviour by testing whether its performance decreases as attention is applied to earlier convolutional layers.

The updating of the particle filter in SPA operates on a much shorter time scale compared to the training of the deep neural network components via backpropagation. This reflects the problem faced by the brain as learning in the visual stream is slow and so the brain needs to use attention to rapidly pick from pre-existing representations in order to solve the current task. However, gradual learning does still occur in the visual stream and is thought to underlie the gradual change from novice to expert on perceptual categorisation tasks (Schyns et al., 1998). From the perspective of SPA, gradual learning in VGG-16 could lead to the emergence of new features for the particle filter to attend to and for the Deep RL algorithm to utilise. As the learning would be slow and occur over several tasks, these new features would generalise over the tasks being repeatedly performed by the agent. Observed behaviour then becomes an interaction between two processes occurring at very different time scales: (1) the slow emergence of new features predictive of temporally stable elements in the environment, and (2) the rapid selection of existing features driven by immediate (or short term) reward. Future work should therefore explore whether slow incremental learning in VGG-16 would improve the performance of SPA over a continuous sequence of different tasks.

The weights of VGG-16 are the result of extensive pre-training on an image classification task known as Imagenet (Deng et al., 2009). Importantly, the data that was used to pre-train VGG-16 involved images that were not used for the multiple choice task and the object collection game. In theory, using the original Imagenet dataset that it was trained on may have lead to better results in the multiple choice task. This is because VGG-16 will have already been trained to produce feature values that distinguish between the object categories in Imagenet, making it easier for SPA to attend to discriminating sets of features. This hypothesis is supported by our analysis of the features produced by VGG-16 during the multiple choice task. For the combination of image categories that SPA performed best on, the vectors of mean feature values were further apart in Euclidean space compared to the combination of image categories that SPA performed worst on. This suggests that the more dissimilar the feature values are between the different image categories, the easier it is for SPA to discriminate between them and quickly change its focus of attention.

This dependence of SPA on the properties of the underlying representations that it attends to opens up several interesting avenues of future research. In particular, it would be interesting to explore whether the use of disentangled representations (Higgins et al., 2018, Higgins et al., 2016) could further improve performance. Independent factors of variation may be easier to attend to because the informative features are separate from each other and so only simple hypotheses are required to solve the task at hand rather than complex combinations of features. Another major benefit of disentangled representations would be that the resulting attention vector would be more interpretable as each attended feature has a natural interpretation; e.g., colour or shape.

## 6 Concluding remarks

We have presented a novel algorithm, termed Selective Particle Attention (SPA), that rapidly and flexibly identifies subsets of latent features for a downstream Deep Reinforcement Learning (RL) algorithm. SPA is a general approach that is independent of the method used to extract the latent representations or the Deep RL algorithm that is applied to them, so long as the Deep RL algorithm approximates a value function. Using two tasks as a proof-of-concept, we show that SPA greatly improves the efficiency and flexibility of learning compared to approaches that use all of the latent features for Deep RL or that use backpropagation to attend to specific features. At its core, SPA uses a particle filter to infer which features are relevant for the current task using only reward feedback. Crucially, this particle filter uses approximate inference instead of backpropagation and so it can be updated rapidly and dynamically. The inference process is based on the magnitude of activation of the features and how well they predict future reward, which is inspired by theories from neuroscience. Future work should explore how the nature of the latent representations affects the behaviour of SPA. For example, does SPA benefit from attending to latent representations that are increasingly abstract or disentangled, and can they be learned slowly over many tasks to complement the fast learning of SPA?

## Declaration of Competing Interest

The authors declare that they have no known competing financial interests or personal relationships that could have appeared to influence the work reported in this paper.

## Figures and Tables

**Figure:**
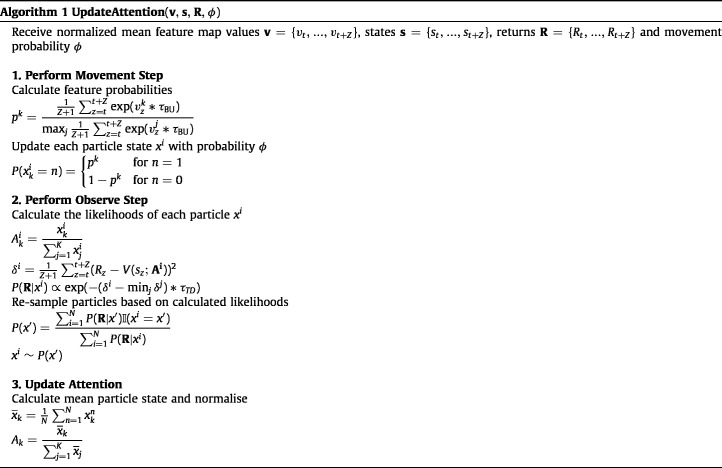


## Appendix

### A.1 Full algorithm for updating attention

Algorithm 1 shows the full pseudo-code for updating the attention vector A. The input consists of vectors of past normalised mean feature map values, states and estimated returns from time t to t+Z, as well as the movement probability ϕ. For the multiple choice experiments Z was equal to 0 and for the object collection game it was equal to 9. A value of 9 was chosen for the object collection game because it corresponded to the length of the n-step updates used in the Deep RL algorithm (see Appendix A.3). By averaging over multiple time-steps in Algorithm 1, the variance of the feature probabilities and the likelihood of each particle state are reduced.

### A.2 Full algorithm for the multiple choice task

For the multiple choice task we passed the results of the attention layer to a fully connected three layer feed forward Deep Neural Network (DNN) (512 units, 256 units and 1 unit). The DNN was used to approximate the value of a given state, which in this case was the value of a given image. To select an action the value of each image was calculated and one of the images was chosen in an ε-greedy manner, with ε=.2. As each trial was based on a single choice, the problem is equivalent to a single step Markov Decision Process (MDP). We therefore trained the DNN after each trial to minimise the squared error between the reward experienced on a trial ($r_{t}$) and the predicted value of the chosen image ($s_{t}$):

(14) $$J\left(\theta \right)=\frac{1}{2}{\left(r_{t}-V\left(s_{t};\theta ,A\right)\right)}^{2}$$

(15) $$\nabla_{\theta }J\left(\theta \right)=-\left(r_{t}-V\left(s_{t};\theta ,A\right)\right)\nabla_{\theta }V\left(s_{t};\theta ,A\right)$$

 where θ are the parameters of the DNN, A is the attention vector, $s_{t}$ is the image chosen at time t and $r_{t}$ is the reward associated with the image chosen. We used RMSProp as an optimiser and the hyper-parameter values can be found in Table 1. For the particle filter of SPA, the return ($R_{t}$) in Eq. (8) was simply the reward experienced at the end of each trial ($r_{t}$).

### A.3 Full algorithm for the object collection game

For the object collection game we passed the results of the attention layer to a fully connected layer (1024 units) followed by a Long Short-Term Memory (LSTM) (Hochreiter & Schmidhuber, 1997) network (256 units), which output the state value (1 unit) and a softmax distribution over actions (2 units). The network therefore resembled an advantage actor–critic (A2C) architecture (Mnih et al., 2016), whereby the predicted state value from the critic was used to calculate the advantage function. We chose this setup to explore whether SPA could work when the network also had to compute a policy. The agent received every 8th frame as input. The cost function of the critic was the mean squared error between the return and the predicted value:

(16) $$J\left(\theta \right)=\frac{1}{2}{\left(R_{t}-V\left(s_{t};\theta ,A\right)\right)}^{2}$$

(17) $$\nabla_{\theta }J\left(\theta \right)=-\left(R_{t}-V\left(s_{t};\theta ,A\right)\right)\nabla_{\theta }V\left(s_{t};\theta ,A\right)$$

 where θ are the parameters of the DNN, A is the attention vector, $s_{t}$ is the frame of the game at time t and $R_{t}$ is the return from state $s_{t}$. The actor was updated using the advantage calculated from the critic:

(18) $$A\left(s_{t},a_{t}\right)=R_{t}-V\left(s_{t};\theta ,A\right)$$

(19) $$\nabla_{\theta }L\left(\theta \right)=A\left(s_{t},a_{t}\right)\nabla_{\theta }\ln\pi \left(a_{t}|s_{t};\theta ,A\right)+\beta \nabla_{\theta }H\left(\pi \left(a_{t}|s_{t};\theta ,A\right)\right)$$

 where H represents the entropy of the softmax distribution over actions and is included to encourage exploration. β is a free parameter that controls the strength of this entropy term and was set to 0.01 for all simulations. In all cases, the return $R_{t}$ from state $s_{t}$ was estimated using n-step Temporal Difference (TD) learning:

(20) $$R_{t}^{n}=\overset{n-1}{\underset{i=0}{\sum }}\gamma^{i}r_{t+i}+\gamma^{n}V\left(s_{t+n};\theta ,A\right)$$

This was used for calculating the target for the critic via Eq. (16) and for calculating the advantage in Eq. (18). The training procedure was the same as in Mnih et al. (2016) with $t_{max}$ set to 10. More specifically, the agent selects actions up until $t_{max}$ or the trial ends. At this point the gradients are calculated for each of the n-step TD-learning updates using the longest possible n-step return. For example, the update for the last state will be a one-step update whereas the update for the first state will be a $t_{max}$-step update. These gradients are all applied in a single update.

The returns calculated using the aforementioned procedure were also used in Eq. (8) for calculating the observation step of the particle filter. However, the movement and update steps of the particle filter were not performed on every training step (when $t_{max}$ or the trial ends). Instead they were performed every $c_{max}$ training steps, we found that this improved both stability and reduced training time. The values used for the hyper-parameters can be found in Table 1.
